# Screening for the causes of refractory hypoxemia in critically ill patients: A case report

**DOI:** 10.3389/fmed.2022.1065319

**Published:** 2022-12-12

**Authors:** Wanglin Liu, Xin Ding, Huaiwu He, Yun Long, Na Cui

**Affiliations:** Department of Critical Care Medicine, Peking Union Medical College Hospital, Chinese Academy of Medical Science and Peking Union Medical College, Beijing, China

**Keywords:** hypoxeima, causes, lung ultrasound, echocardiography, electrical impedance tomography

## Abstract

Hypoxemia was a very common symptom in critical patients and should be treated immediately before resulting in permanent organ failure. Rapid diagnosis of the etiology of hypoxemia could be achieved by combining the use of various bedside and radiation-free techniques such as lung ultrasound, electrical impedance tomography and echocardiography. By presenting a case of serious acute refractory hypoxemia, we proposed an efficient protocol for diagnosing and treating hypoxemia in a safe and fast way.

## Introduction

Hypoxemia is defined as a low oxygen level in the blood. It is most commonly seen in critically ill patients and is a very common reason for admitting to intensive care unit (ICU). Hypoxemia could cause tissue hypoxia and lead to organ failure and life-threatening conditions (1). There are various causes of hypoxemia including hypoventilation, lung ventilation/perfusion mismatch, right-to-left shunt, diffusion impairment, and low inspired partial pressure of oxygen (2). Identifying the right cause and correcting hypoxemia in time is crucially important for critical patients. The authors present a case of middle-aged patient who developed unusual acute and severe hypoxemia after receiving a congenital atrial defect repair surgery. A series of assessments were performed for this patient to identify and treat the severe hypoxemia.

## Case report

A 41-year-old man diagnosed with congenital atrial septal defect was admitted to our surgical ICU after an atrial defect repair surgery through right lateral thoracotomy approach. He had received jejunal stromal tumor surgery 1 year ago, when the congenital atrial defect was found by routine echocardiography. The patient had no other notable past medical history. He was asymptomatic before the cardiac surgery. At admission to the cardiac surgery ward before surgery, he showed a pulse oxyhemoglobin saturation (SpO2) of 96.6%, arterial partial pressure of oxygen (PaO2) of 79 mmHg by arterial blood gas (ABG) analysis. Preoperative transthoracic echocardiography (TTE) showed an atrial septal defect size of 36 mm near the inferior vena cava with left to right shunt flow and estimated systolic pulmonary artery pressure of 38 mmHg.

The operation lasted about 8 h. ABG drawn before intubation and mechanical ventilation showed: pH 7.37, arterial partial pressure of carbon dioxide (PaCO2) 35.6 mmHg, PaO2 115 mmHg, base excess (BE) −3.6 mmol/L, lactate 0.9 mmol/L. The ventilation strategy used during the surgery was as follows: volume control, tidal volume (VT) of 450 ml, respiratory frequency (f) of 13 breaths/min, fraction of inspired oxygen (FiO2) of 100%, positive end-expiratory pressure (PEEP) of 5 cmH2O. ABG drawn at the end of the operation showed: pH 7.32, PaCO2 50.1 mmHg, PaO2 91.7 mmHg, BE −0.8 mmol/L, lactate 1.3 mmol/L. Routine intraoperative TEE conducted by the anesthetist at the end of the surgery did not reveal significant abnormal structure cardiac defect. The cardiac surgeon who performed the surgery claimed the operation successful. The patient was transferred from the operation room to ICU at midnight under sedation and intubation for post-cardiac surgery care. He presented with a hear rate (HR) of 100 b.p.m, blood pressure (BP) of 150/93 mmHg, SpO2 of 93% (mechanical ventilation, volume control, FiO2 of 50%, VT of 450 ml, PEEP of 8 cmH2O, f of 15 breaths/min). ABG showed a PaO2 of 58 mmHg, arterial partial pressure of carbon dioxide (PaCO2) of 47 mmHg. After adjusting the ventilator settings (volume control, FiO2 100%, VT 500 ml, PEEP 8 cmH2O, f 18 breaths/min), ABG was drawn again and showed a PaO2 of 81 mmHg, PaCO2 of 31 mmHg. The patient was severely hypoxic with a P/F ratio < 100 mmHg. In order to figure out and correct the cause of hypoxemia, we performed a series of examinations in terms of airway, lung ventilation, lung perfusion, and ventilation/perfusion (V/Q) mismatch.

## Airway check

Firstly, there was no obstruction in the airway, as we made sure that there was neither mucus plugging nor airway stenosis. There was no air leak in the artificial airway and the breathing circuit. Hypoventilation was ruled out since the PaCO2 returned to normal after adjusting the ventilator, with minute ventilation volume about 9 L/min. Thus, airway related etiologies of hypoxemia were ruled out in the first place.

## Lung ultrasound and electrical impedance tomography ventilation

**Figure 1:**
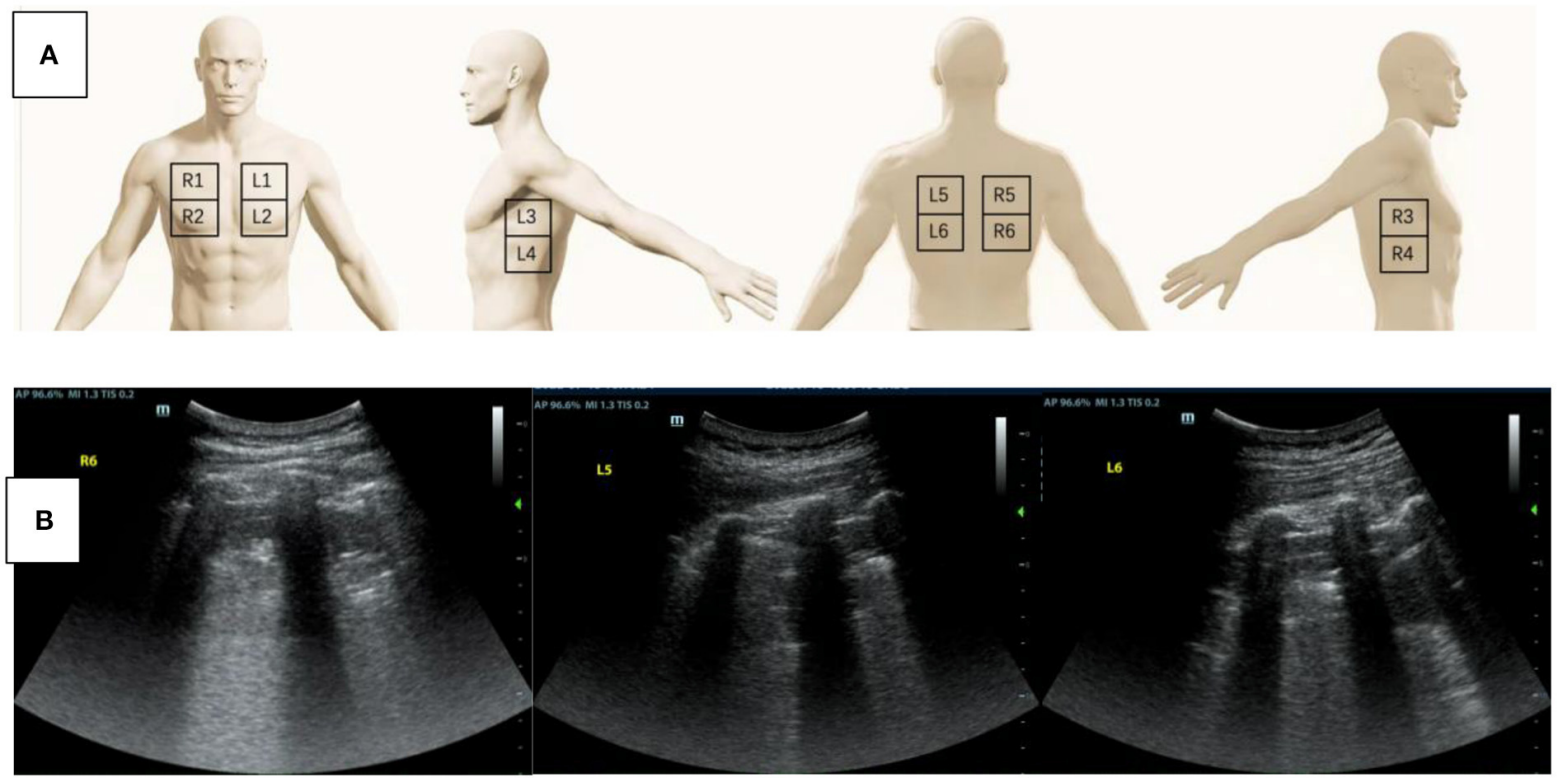
**(A)** A schematic diagram of the twelve-zone scanning lung ultrasound protocol. **(B)** Lung ultrasound was performed to assess the cause of hypoxemia. Tissue-like signs were found in the region of R6, L5, and L6.

**Figure 2:**
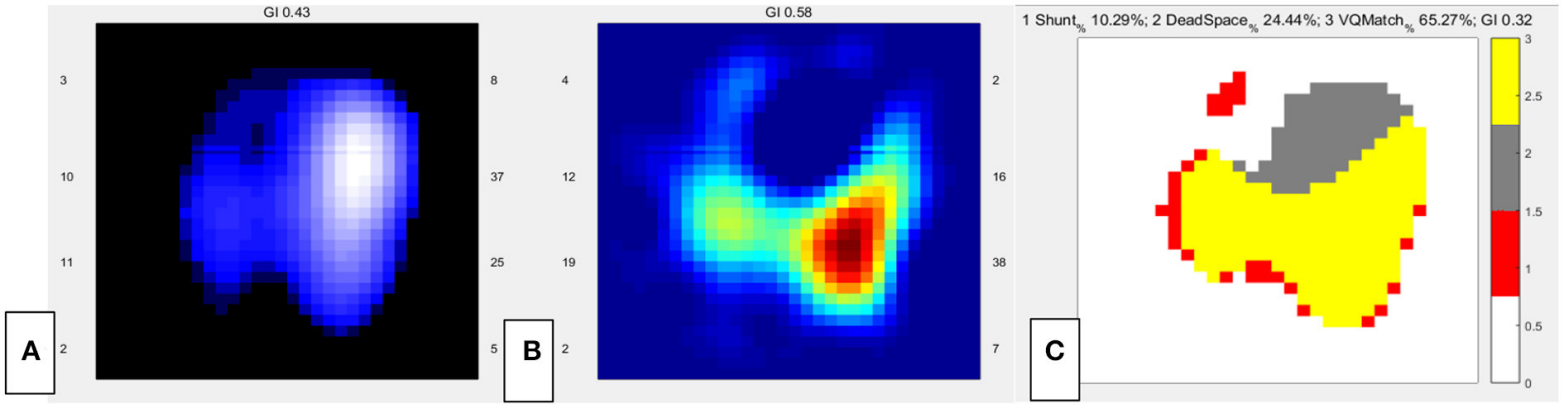
EIT images of the functional ventilation distribution **(A)** (dark blue areas indicated low ventilated regions and white areas indicated high ventilated regions), functional perfusion distribution **(B)** (red areas indicated high-perfusion regions and blue areas indicated low-perfusion regions), and distribution of the regional ventilation/perfusion ratios **(C)** (Ventilated regions were defined as pixels with impedance changes higher than 20% of the maximum tidal impedance variation in the functional ventilation image. Perfused regions were defined as pixels higher than 20% of the maximum bolus-related impedance change in the functional perfusion image. Gray areas indicated regions with high ventilation and low perfusion. Red areas indicated low ventilation and high perfusion regions. Yellow areas indicated good ventilation-perfusion matching).

Was lung collapse or inhomogeneous ventilation the cause of hypoxemia in this patient? We performed a quick bedside lung ultrasound using a twelve-zone scanning protocol (3) to figure out whether lung consolidation or pleural effusion existed. The result showed tissue-like signs, which indicated lung consolidation, in the regions of R6, L5, and L6 (Figure 1), while lung sliding and A-lines were observed in the other regions. Pulmonary edema was also not supported by lung ultrasound findings. The chest X ray was not remarkable except for decreased transparency in the right lower lung field. To improve hypoxemia that could be caused by the consolidation in the dorsal regions of bilateral lungs, lung recruitment maneuver with PEEP of 20 cmH2O and peak airy way pressure of 40 cmH2O for 2 min was performed, but little effect was achieved. To check for regional ventilation distribution, we then conducted bedside electrical impedance tomography (EIT) (4). EIT measurements were performed with PulmoVista 500 (Dräger Medical, Lübeck, Germany). A silicone EIT belt with 16 surface electrodes was placed around the patient's thorax at the fourth intercostal space level. EIT measurements were continuously recorded at 20 Hz when the patients were at relative stable condition after medical treatment (5). The EIT ventilation image of our patient showed decreased ventilation distribution in the right lung (Figure 2A), which was probably related with the right lateral thoracotomy surgical approach. Decreased right lung ventilation might contributed partly to hypoxemia in this patient. But the main cause still remained to be detected.

## EIT Perfusion

Was V/Q mismatch the main cause of hypoxemia in this patient? We reviewed the past medical history and found that lung V/Q mismatch related comorbidities including interstitial lung disease and chronic obstructive pulmonary disease were not present in our patient. And preoperative TTE showed that pulmonary hypertension was not significantly increased. However, newly developed disease conditions such as lung atelectasis and vascular embolism could also lead to V/Q mismatch. Thus, we decided to assess lung V/Q match by EIT. After obtaining informed consent, 10 ml of hypertonic saline (10%) bolus infusion was performed at bedside to assess the regional pulmonary ventilation and perfusion mismatch (5–7). EIT data were recorded throughout the PEEP titration in the supine position. During this period, the patient was fully sedated using continuous infusion of midazolam, propofol and fentanyl to prevent any spontaneous breathing (5). The data of V/Q match were analyzed offline using customized software programmed with MATLAB R2015 (the MathWorks Inc., Natick, MA) (5). Perfusion of the right lung was markedly low. The dead space of the whole lung was 24.4%, and the shunt was 10.29% (Figures 2B,C).

## CTPA

**Figure 3:**
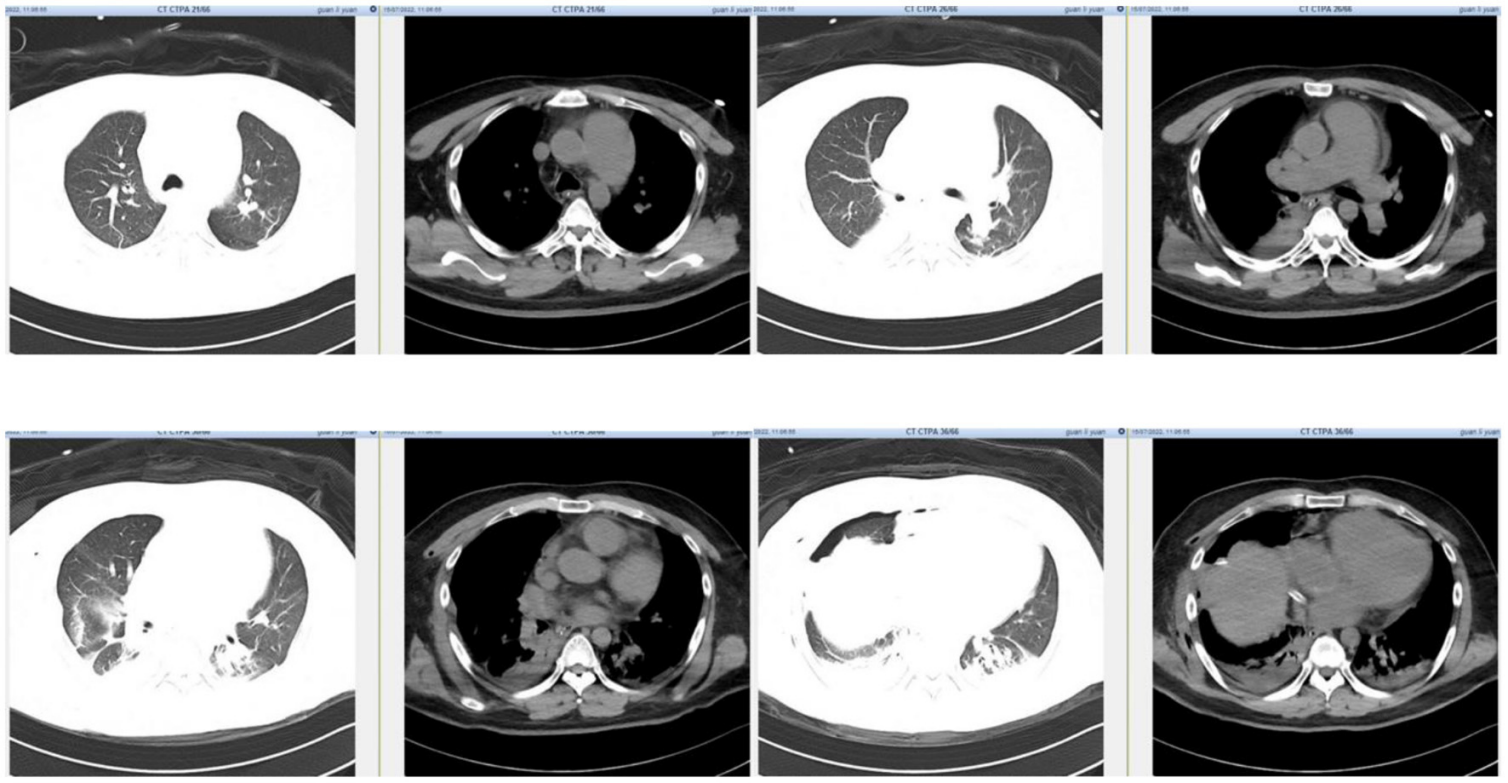
CTPA demonstrated mild atelectasis in the inferior lobes of bilateral lungs and a small amount of operation related pneumothorax. The small embolism in the superior lobar branch of right pulmonary artery was not shown in the picture.

Our previous study found a cutoff value of 30.37% dead space was related to pulmonary embolism (PE). However, the relatively high dead space ventilation together with a history of jejunal stromal tumor might be indicative of potential pulmonary embolism in this case. But bedside ultrasound examination did not find venous thrombosis in bilateral lower extremities. With the aim to exclude pulmonary embolism, we transferred the patient for a computed tomography pulmonary angiography (CTPA) in the following morning. CTPA revealed small embolism in the superior lobar branch of the right pulmonary artery (Figure 3). The main pulmonary trunk was markedly widened, but not significantly changed compared with the preoperative CT scan. There was mild atelectasis in the inferior lobes of bilateral lungs and a small amount of operation related pneumothorax as showed in the CTPA. The poor perfusion of the right lung by EIT was consistent with the result of CTPA. However, neither the mild atelectasis nor the small embolism in the superior lobar branch of right pulmonary artery were able to explain the severity of refractory hypoxemia in this patient in our experience.

## Transesophageal echocardiography

**Figure 4:**
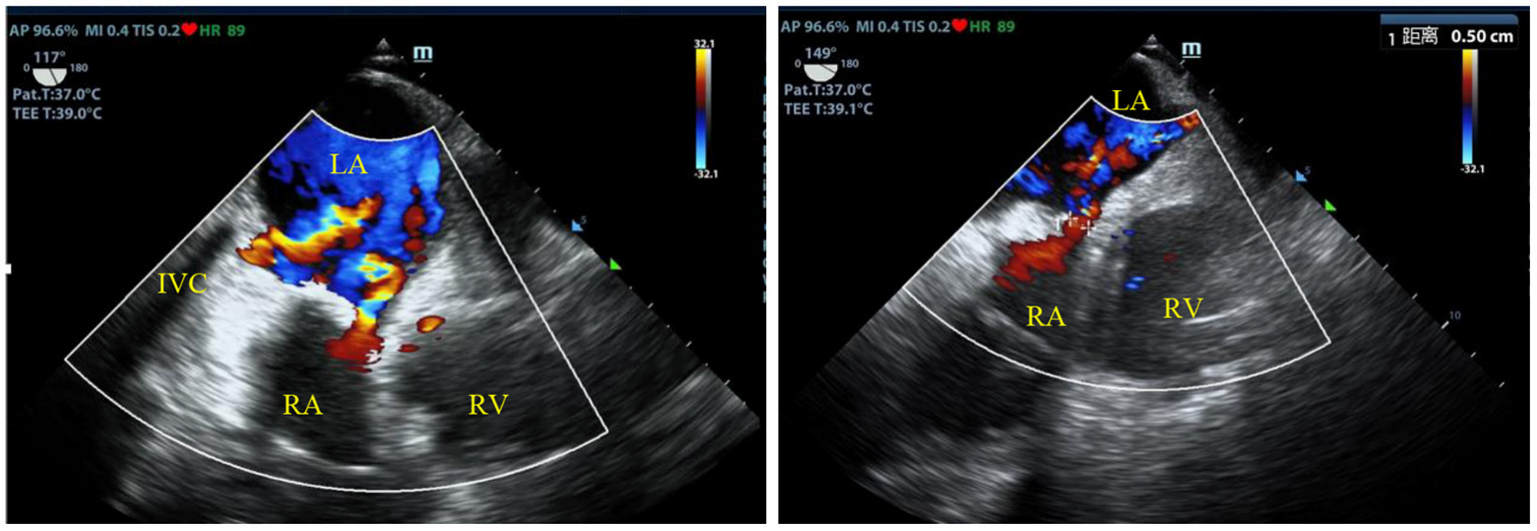
TEE showed a residual atrial septal defect with a size of 5 mm and bidirectional multicolored shunt flow signal. And an IVC-left atrium shunt was also found soon after the repair. TEE, transesophageal echocardiography; LA, left atrium; RA, right atrium; RV, right ventricle; IVC, inferior vena cava.

The cardiac structure and function were urgently to be examined, since the main cause of hypoxemia still remained unclear in our patient. Pulmonary arteriovenous malformation or potential right to left intracardiac shunt resulted by abnormal cardiac structure might be the last cause of severe hypoxemia in this case. Hypoxemia caused by intracardiac shunt usually responded poorly to increase of inspired oxygen concentration, as in the case with our patient. TTE image was not clear because of the influence of surgical operation. We thus conducted bedside transesophageal echocardiography (TEE). Finally, we found a residual atrial septal defect with a size of 5 mm by TEE. Bidirectional multicolored shunt flow signal was detected through this atrial defect (Figure 4). In addition, TEE revealed a second shunt flow signal through a defect possibly between the inferior vena cava (IVC) and the left atrium. After all, the intracardiac shunt found by TEE seemed to be able to explain the refractory hypoxemia in this patient.

## Treatment and outcome

After the above examinations, we concluded that the residual right-left atrial shunt and the IVC-left atrial shunt most likely had the biggest impact on this patient's severe hypoxemia. The cardiac surgeon scheduled a repair of the surgery. During the patient's waiting period for a second surgery, we conducted intermittent prone positioning to improve ventilation distribution of the dependent-dorsal lung regions. EIT has been used in perioperative patients for individual optimization of ventilator settings (8). For post-operative patients who underwent a long operation, the risk of developing acute respiratory disease syndrome was high. The patient presented mild atelectasis in the inferior lobes of bilateral lungs showed by lung ultrasound and CTPA. Thus, we titrated PEEP by EIT with the crossing method, which is routinely conducted by us in our department (6). During the process of PEEP titration using EIT, we closely monitored the patient's hemodynamics to avoid severe adverse hemodynamic effect. The patient was hemodynamically stable without any vasoactive agents during the process. Thus, we set an optimal PEEP of 9 cmH2O based on the result of EIT PEEP titration for better gas distribution in the lungs. Esophageal pressure and other respiratory mechanics were monitored to avoid patient self-inflicted lung injury (P-SILI). However, hypoxemia progressively deteriorated to PaO2 of 47 mmHg at ventilator FiO2 of 100% (P/F ratio = 47 mmHg) on the third day after the first surgery, when a second surgery was scheduled. The surgeons confirmed the residual IVC-left atrium and the atrial septal defect during the second open-heart surgery through the midline sternotomy approach. They removed the originally mis-sutured patch and re-patched the defect. Hypoxemia was greatly improved on the first night after the second surgery, with a PaO2 of 134 mmHg at ventilator FiO2 of 60% (P/F ratio = 223 mmHg). With pulmonary physical therapy and negative fluid balance, the P/F ratio further improved (P/F ratio = 322 mmHg) at the third day after the second surgery. The patient was off the ventilator and extubated successfully 3 days later. He was transferred back to the general ward the day after extubation and subsequently discharged from the hospital.

## Discussion

Post-operative hypoxemia is very common in ICU patients who had underwent thoracic or cardiac surgery. Various etiologies including interstitial lung disease, COPD, pulmonary hypertension, pulmonary embolism, pulmonary edema, pneumonia, atelectasis, mucus plugging, pulmonary arteriovenous malformation, and right to left intracardiac shunt could be the cause. These etiologies cause hypoxemia through mainly three mechanisms: impaired diffusion, V/Q mismatch and shunt. In our case, the patient developed severe hypoxemia soon after cardiac surgery. Through rapid bedside evaluation, we ruled out airway obstruction, hypoventilation, pulmonary edema, and extreme lung V/Q mismatch. The small PE revealed by CTPA could also not explain the severe hypoxemia in our case. Finally, intracardiac shunt found by TEE was confirmed to be the main cause of hypoxemia in this patient. A second repair surgery successfully corrected the cause and cured the patient.

Treating the patient with acute life-threatening hypoxemia during mechanical ventilation should begin with a quick and safe approach, with the goal of improving oxygenation and minimizing the harmful effects. There are various assessments to investigate the causes of hypoxemia in clinical practice. For non-ICU patients, CT or CTPA when necessary is most often conducted. For ICU patients who are in critical conditions, transferring to a CT machine for a scan is risky and may even be life-threatening. The development of new techniques including lung ultrasound and EIT enabled quick and convenient diagnosis of hypoxemia at bedside without the risk of transferring a critical patient. EIT is a non-invasive technique that provides dynamic tidal images of gas distribution (9). EIT has been widely used for lung recruitment, positive end-expiratory pressure (PEEP) adjustment, lung volume estimation, and homogeneity of gas distribution during mechanical ventilation (9). The combination of these new techniques (EIT, lung ultrasound) and the traditional imaging methods (chest X-ray, CTPA) could provide quicker, better and more accurate diagnostic power for identifying causes of hypoxemia. Undoubtedly, this could lead to better outcome for the critical patients.

Lung ultrasound can be performed conveniently and repeatedly at bedside (10), allowing immediate diagnosis of different lung pathologies including lung consolidation, interstitial disease, pneumothorax, and pleural effusion at any emergent conditions and free of radiations. Lung sliding and A-lines were normal dynamic and static signs by lung ultrasound. The quad or sinusoid sign by lung ultrasound indicated pleural effusion. Tissue like sign and the shred sign indicated alveolar consolidation. B-lines by lung ultrasound were indicative of interstitial syndrome caused either by hemodynamic or inflammatory pulmonary edema (10). By combining the use of lung ultrasound and echocardiography, hemodynamic pulmonary edema could be diagnosed and treated timely. Pneumothorax could be detected as lung point sign or absence of lung sliding by lung ultrasound before confirming by chest X-ray. Lung consolidation or atelectasis can be detected by lung ultrasound and further verified by decreased ventilation of the dorsal lung in functional EIT ventilation image.

EIT could provide both lung ventilation and perfusion image (4, 11). Pneumothorax could also present as decreased regional ventilation and could be supported by lung point sign in lung ultrasound. Ventilation defect of gravity dependent area detected by EIT could be caused by lung consolidation, which presented as tissue like or shred sign in lung ultrasound. The ventilation/perfusion match image by EIT would reveal increased shunt flow when lung consolidation was the main cause of hypoxemia. Both ventilation and perfusion of the right lung was decreased by EIT in our patient. As a result, the shunt was relatively low (10.29%). Hypoxic pulmonary vasoconstriction might have contributed to decreased perfusion by compensatory mechanism. And the small embolism in the superior lobar branch of the right pulmonary artery might also have contributed to decreased perfusion. Further experimental research is warranted to validate these mechanisms. Increased dead space by EIT indicated pulmonary vascular dysfunction, among which PE was the most common etiology. Increased dead space by EIT enhanced the indication for performing CTPA and might decrease unnecessary risk for transferring the critical patient.

In our case, we have ruled out all common causes of hypoxemia after clearing airway and breathing circuit and completing lung ultrasound, EIT and CTPA in the early stage. there was one rare but non-negligible cause, intracardiac shunt, remained to be examined. The patient developed newly unexplained refractory hypoxemia soon after atrial defect repair surgery, which might imply cardiac causes of hypoxemia. Echocardiography (TEE or TTE) was the right assessment for cardiac causes. The residual right-left atrial shunt and the IVC-left atrial shunt revealed by the TEE proved to be the prime cause of refractory hypoxemia in our case, confirmed by the second open-heart surgery. Previous literature reported complication of hypoxemia soon after the repair surgery of inferior sinus venosus defects, which is a rare type of interatrial communication involving lower part of the atrial septum derived from the sinus venosus. Preoperative diagnosis of this type of atrial defect is difficult and challenging (12). The lower edge of the defect has no residual atrial septal tissue thus the orifice of IVC strides over the atrial septum. Giant residual Eustachian valve in some patients may be mistaken as the lower edge of the defect, leading to mis-suture in the surgery and resulting in residual IVC- left atrial shunt. The patient in our case fell into this situation. TEE proves to be better than TTE in accurately diagnosing this type of defect because of close proximity of atrial septum to TEE transducer. TEE is the diagnostic procedure of choice and should be suggested when atrial defect near IVC was identified before operation or at any time. However, diagnosing inferior sinus venosus defects by TTE can still be difficult in adult patients with congenital heart disease. Contrast echocardiography with microbubbles provides accurate detection of cardiovascular shunts. However, it fails to reveal detailed information about anatomical and functional characterizations. Cardiac MRI can also be used to precisely determine ventricular volumes, degree of left-to-right shunts, as well as anatomical vascular and cardiac abnormalities. Contrast-enhanced CT scan is another valuable method for detection of the defect. But this method leads to exposure to ionizing radiation and may reveal insufficient information (13). Furthermore, intraoperative injection of agitated saline contrast *via* intravenous cannulation in a lower extremity can allow for detection and correction of residual defects during the operation. Careful intraoperative examination of the posterior-inferior portion of the interatrial septum should be done in patients with isolated interatrial shunts and in patients with concomitant congenital heart defects (14). Accurate preoperative diagnosis is critical for post-operative recover of the patient. Careful preoperative planning and intraoperative examination could significantly reduce the incidence of residual defects and free the patient from reoperation (14).

**Figure 5:**
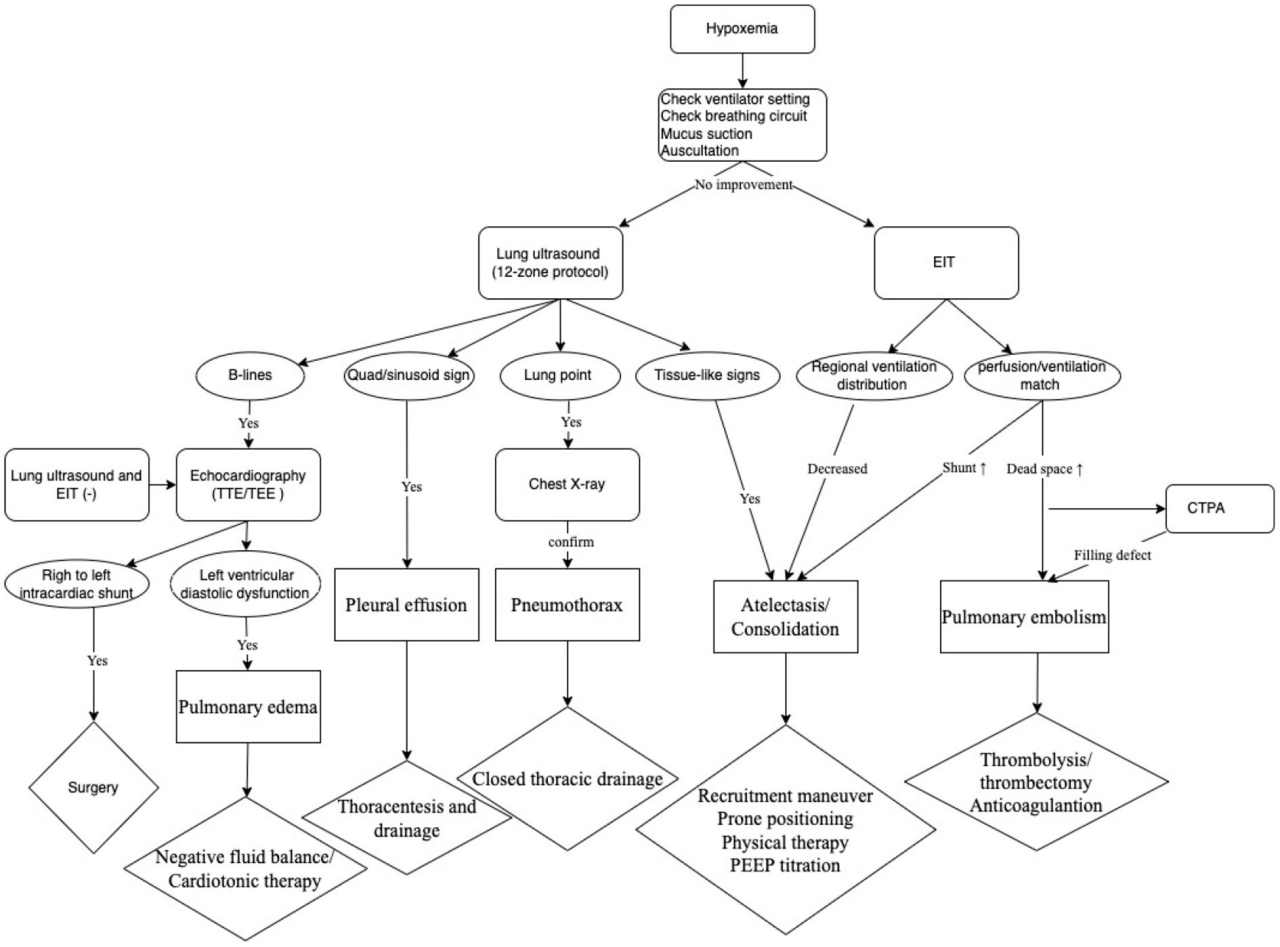
The protocol for assessments of hypoxemia in ICU critical patients.

Bedside technologies including lung ultrasound, EIT ventilation and perfusion image, and echocardiography were combinedly used in our patient. Based on the clinical practice, we developed a protocol, which consisted of a bundle of assessments, to screen for etiology and treat newly developed acute hypoxemia in critical patients in our ICU (Figure 5). In our protocol, all potential causes for hypoxemia could be detected.

## Conclusion

In conclusion, we developed a protocol of assessments for rapidly identifying etiologies of hypoxemia. Thus, safe and timely treatment was expected to be achieved by our protocol in ICU critically ill patients. The protocol combined various methods and technologies, mainly lung ultrasound, echocardiography and EIT, which could be conveniently conducted at bedside, reducing medical risk by shortening the time to diagnosis and free of transportation in patients with life-threatening conditions. This protocol could assist intensivist to better diagnose and treat hypoxic critical patients.

## Data availability statement

The studies involving human participants were reviewed and approved by Peking Union Medical College Hospital.

## Ethics statement

Written informed consent was obtained from the next of kin of the patient for the publication of any potentially identifiable images or data included in this article.

## Author contributions

WL and XD: collected data and drafted the manuscript. NC, HH, and YL: revised the manuscript. All authors have read and approved the final version of the manuscript.
